# Chronic Malaria Is Associated With Trauma-related Splenic Rupture Requiring Splenectomy

**DOI:** 10.1093/infdis/jiaf629

**Published:** 2025-12-11

**Authors:** Putu A I Shanti, King Alexander, Freis Candrawati, Benediktus Andries, Noy Norman Kambuaya, Hasrini Rini, Aisah R Amelia, Agatha M Puspitasari, Ristya Amalia, Desandra A Rahmayenti, Leo Leonardo, Pak Prayoga, Leily Trianty, Zuleima Pava, Enny Kenangalem, Sarah Auburn, Ric N Price, Ida Safitri Laksanawati, Pierre A Buffet, Rintis Noviyanti, Nicholas M Anstey, Jeanne R Poespoprodjo, Steven Kho

**Affiliations:** Surgery Department, Mimika District Hospital, Timika, Papua, Indonesia; Surgery Department, Mimika District Hospital, Timika, Papua, Indonesia; Papuan Health and Community Development Foundation, Timika Laboratory Facility, Timika, Papua, Indonesia; Papuan Health and Community Development Foundation, Timika Laboratory Facility, Timika, Papua, Indonesia; Papuan Health and Community Development Foundation, Timika Laboratory Facility, Timika, Papua, Indonesia; Papuan Health and Community Development Foundation, Timika Laboratory Facility, Timika, Papua, Indonesia; Exeins Health Initiative, Malaria Pathogenesis, Jakarta, Indonesia; Exeins Health Initiative, Malaria Pathogenesis, Jakarta, Indonesia; Exeins Health Initiative, Malaria Pathogenesis, Jakarta, Indonesia; Papuan Health and Community Development Foundation, Timika Laboratory Facility, Timika, Papua, Indonesia; Papuan Health and Community Development Foundation, Timika Laboratory Facility, Timika, Papua, Indonesia; Papuan Health and Community Development Foundation, Timika Laboratory Facility, Timika, Papua, Indonesia; Eijkman Research Centre for Molecular Biology, BRIN, Cibinong, Indonesia; Menzies School of Health Research and Charles Darwin University, Global and Tropical Health Division, Darwin, Northern Territory, Australia; Papuan Health and Community Development Foundation, Timika Laboratory Facility, Timika, Papua, Indonesia; Menzies School of Health Research and Charles Darwin University, Global and Tropical Health Division, Darwin, Northern Territory, Australia; Menzies School of Health Research and Charles Darwin University, Global and Tropical Health Division, Darwin, Northern Territory, Australia; Centre for Tropical Medicine and Global Health, Nuffield Department of Clinical Medicine, University of Oxford, Oxford, United Kingdom; Mahidol-Oxford Tropical Medicine Research Unit, Faculty of Tropical Medicine, Mahidol University, Bangkok, Thailand; Centre for Child Health-PRO, Universitas Gadjah Mada, Yogyakarta, Indonesia; Institut Pasteur, University of Paris, Paris, France; Eijkman Research Centre for Molecular Biology, BRIN, Cibinong, Indonesia; Menzies School of Health Research and Charles Darwin University, Global and Tropical Health Division, Darwin, Northern Territory, Australia; Papuan Health and Community Development Foundation, Timika Laboratory Facility, Timika, Papua, Indonesia; Centre for Child Health-PRO, Universitas Gadjah Mada, Yogyakarta, Indonesia; Pediatrics Department, Mimika District Hospital, Timika, Papua, Indonesia; Papuan Health and Community Development Foundation, Timika Laboratory Facility, Timika, Papua, Indonesia; Menzies School of Health Research and Charles Darwin University, Global and Tropical Health Division, Darwin, Northern Territory, Australia

**Keywords:** chronic asymptomatic malaria, trauma, spleen rupture, splenectomy

## Abstract

Splenic rupture is a recognized complication of acute *Plasmodium falciparum* and *Plasmodium vivax* malaria, but the risk of splenic rupture in chronic asymptomatic infections is unknown. In Timika, Papua, Indonesia, we determined the proportion of PCR-detectable asymptomatic peripheral parasitemia in patients undergoing trauma-related splenectomy (2015–21) and found it was more than twice the proportion compared to a 2013 household survey of the general population (87.9% [29/33] vs 38.6% [697/1807]; *P* < .0001). Our findings suggest asymptomatic parasitemia with either *P. falciparum* or *P. vivax* is associated with splenic rupture following trauma, pointing toward an additional consequence of chronic infection in malaria-endemic areas.

## Teaser

Asymptomatic *Plasmodium* falciparum or *P. vivax* parasitemia is over twice as frequent in trauma-related splenectomy patients than in the general population in Timika, Indonesia, suggesting that chronic malaria may increase vulnerability to splenic rupture following trauma in malaria-endemic areas.

In malaria-endemic regions, repeated exposure to *Plasmodium* spp. leads to many residents having persistent “asymptomatic” infections (chronic malaria) [1]. These afebrile infections make up a large proportion of global parasite burden [1] and contribute significantly to disease transmission [2]. While the clinical implications of acute symptomatic malaria are well established [3], the long-term consequences of chronic subclinical *Plasmodium* infections remain underappreciated.

Chronic parasitemia is associated with significant complications and poor long-term outcomes [4]. These include recurrent episodes of symptomatic parasitemia, impaired erythropoiesis, and chronic loss of infected and uninfected red blood cells, causing chronic anemia and associated morbidity and mortality [4–6]. There is also an increased risk of invasive bacterial coinfections as well as impaired cognitive development and academic performance in children [4]. In pregnancy, placental malaria is usually asymptomatic but associated with adverse outcomes including miscarriage, preterm delivery, low birth weight, and both maternal and neonatal mortality [4]. Recent studies have also revealed that the majority of parasites in chronic asymptomatic infections are hidden in the spleen, revealing a greater previously unrecognized parasite burden and presenting additional hurdles to malaria elimination [7, 8].

An additional consequence of chronic malaria may be increased vulnerability of the spleen to rupture, following even minor trauma. Although splenic rupture has traditionally been associated with acute symptomatic malaria [9, 10], its relationship to chronic asymptomatic infections is poorly studied. Enlargement of the spleen, a hallmark of both acute and chronic malaria, is associated with red blood cell congestion [6], likely increased intrasplenic tension, and a greater surface area, potentially rendering it more vulnerable to trauma. Pathological changes underlying splenomegaly in both acute and chronic malaria may also compromise splenic integrity [9, 10], further contributing to the risk of trauma-related or pathological splenic rupture and greater bleeding from enlarged spleens.

We examined the association between chronic asymptomatic malaria and trauma-related splenic rupture requiring splenectomy, comparing the prevalence of PCR-detectable peripheral parasitemia in individuals undergoing splenectomy following trauma to that in the general population. Our findings provide population-level evidence supporting a possible contributory role of chronic malaria to trauma-related splenic rupture in endemic areas.

## METHODS

The study was conducted in Timika, Papua, Indonesia, a region with high perennial transmission of primarily *P. falciparum* and *P. vivax* [11]. Participant data from two complementary cohorts were analyzed. The first cohort (Cohort 1), conducted between 2015 and 2021, comprised of any patient undergoing splenectomy at Mimika District Hospital due to splenic injury from trauma, as previously described [6–8]. This hospital is the sole public referral hospital for surgical emergencies in the region. The second cohort (Cohort 2), conducted in 2013, included all participants enrolled into a cross sectional household survey of the general Timika population [11]. Due to the rarity of trauma-related splenectomy in children, children younger than 10 years old were excluded from both cohorts. Presence of circulating parasitemia in both cohorts was determined using standard Giemsa-based light microscopy and a nested *Plasmodium* PCR assay of frozen peripheral blood, as described elsewhere [8, 11]. Chronic asymptomatic infections were defined as individuals with microscopy and/or PCR-detectable *Plasmodium* spp. in peripheral blood, with no fever (<37.5°C) or history of fever in the preceding 24 hours. Patients in Cohort 1 had systematic pre-operative clinical assessment in the emergency department as part of hospital procedures, documenting temperature, and presence of malaria symptoms. These were assessed by the research team directly from clinical charts in patient medical records. Following surgery, patients were also interviewed for any history of malaria symptoms, with none of the patients having fever or reported symptoms consistent with acute malaria in the 24 hours prior to surgery. Parasite counts per microliters of blood were calculated for individuals with patent infections. Clinical, demographic and laboratory data were recorded, including spleen weight and total plasma IgM levels in Cohort1 [8]. Data were analyzed using Graphpad Prism v10 (Dotmatics, MA) and Stata. Categorical variables were compared using the chi-squared test. Spleen weights were log transformed and compared using Welch's t-test. Odds ratios (ORs) and 95% confidence intervals (95% CI) were used to compare population proportions. Adjusted OR (aOR) were calculated after controlling for age and sex using logistic regression. *P-*values *<*.05 were considered statistically significant. The studies for both cohorts were approved by the Human Research Ethics Committees of Northern Territory Health and Menzies School of Health Research, Australia, and Universitas Gadjah Mada, Indonesia. Written informed consent was obtained from all participants and/or guardians.

## RESULTS

Between 2015 and 2021, 36 patients underwent splenectomy of whom two were elective procedures and 34 were for splenic rupture related to trauma. The two elective splenectomies and the only child <10 years were excluded, leaving 33 trauma-associated splenectomies in Cohort 1. In Cohort 2, 2830 individuals from the general Timika population participated in the survey of whom 68 (2.4%) were excluded because they had a fever or history of fever, 937 (33.1%) were excluded because they were younger than 10 years old and 262 (9.3%) were excluded because they did not have microscopy examination or PCR of peripheral blood. In total, 1841 participants were included in the analysis—33 from Cohort 1 and 1807 from Cohort 2.

Compared to Cohort 2, splenectomized individuals in Cohort 1 were younger and were more likely to be male (Table 1). Ruptured spleens had a median weight of 335 g (range: 80–979 g), with motorcycle accidents the primary cause of trauma (52%), followed by blunt physical assault (21%), sharp physical assault and pedestrian injury (both 9%), car accidents (6%), and falling from a height (3%). One individual who suffered trauma (785 g spleen and total plasma IgM level of 1410 mg/dL) also fulfilled the criteria for hyperreactive malarial splenomegaly [8]. Spleen weights were significantly higher in individuals with patent (median 414 g [IQR: 341–605 g]) than subpatent infections (median 279 g [IQR: 159–472 g], *P* = .049).

**Table 1.** Baseline Characteristics and Plasmodium Prevalence

Parameter	Splenectomy Patients (Cohort 1), *n* = 33	General Population (Cohort 2), *n* = 1807	OR (95% CI)	aOR (95% CI)
Age in years (median [IQR]; range)	22 ([19–33.5]; 12–50)	30 ([20–40]; 10–90)	-	-
Sex, n of males (%)	26/33 (78.8)	667/1807 (36.9)	-	-
Ethnicity, n of Papuans (%)	17/33 (51.5)	783/1807 (43.3)	-	-
Spleen weight, grams (median [IQR])	335 (245–450)	-	-	-
All *Plasmodium* species (*P. falciparum, P. vivax, P. malariae,* mixed infections)				
Peripheral-detectable patent and subpatent, n/N (%)	29/33 (87.9)	697/1807 (38.6)	11.5 (4.0–33.0)	10.0 (3.5–28.8)
Peripheral-detectable patent, x/N (%)	16/33 (48.5%)	216/1807 (12.0%)	6.9 (3.5–13.9)	5.7 (2.8–11.7)
^a^Patent parasite count per µL blood (median [IQR])	124 (69–700)	336 (113–1188)	-	-
Peripheral-detectable subpatent, y/N (%)	13/33 (39.4%)	481/1807 (26.6%)	1.8 (.9–3.6)	1.7 (.8–3.4)
*P. falciparum*				
Peripheral-detectable patent and subpatent, n/N (%)	14/33 (42.4)	316/1807 (17.5)	3.5 (1.7–7.0)	3.1 (1.5–6.3)
Peripheral-detectable patent, x/N (%)	10/33 (30.3)	114/1807 (6.3)	6.5 (3.0–13.9)	5.4 (2.5–11.9)
^a^Patent parasite count per µL blood (median [IQR])	149 (77–884)	342 (130–1710)	-	-
Peripheral-detectable subpatent, y/N (%)	4/33 (12.1)	202/1807 (11.2)	1.1 (.4–3.1)	1.0 (.4–3.0)
*P. vivax*				
Peripheral-detectable patent and subpatent, n/N (%)	12/33 (36.4)	322/1807 (17.8)	2.6 (1.3–5.4)	2.6 (1.2–5.3)
Peripheral-detectable patent, x/N (%)	5/33 (15.2)	86/1807 (4.8)	3.6 (1.3–9.5)	3.2 (1.2–8.6)
^a^Patent parasite count per µL blood (median [IQR])	64 (35–195)	330 (89–816)	-	-
Peripheral-detectable subpatent, y/N (%)	7/33 (21.2)	236/1807 (13.1)	1.8 (.8–4.2)	1.8 (.8–4.3)

Abbreviations: 95% CI, 95% confidence interval; aOR, adjusted odds ratio; IQR, interquartile range; OR, odds ratio.

^a^Parasite counts missing for 2 *P. vivax* and 1 *P. falciparum* patent infections in Cohort 2.

In Cohort 1, the proportion of individuals with asymptomatic peripheral *Plasmodium* infections (patent or subpatent) was 87.9% (29/33), more than twice that observed in Cohort 2 (38.6% [697/1807], *P* < .0001; Table 1). Compared to Cohort 2, the OR of having chronic infection in Cohort 1 was 11.5 (95% CI: 4.0–33.0). After controlling for age and sex, the aOR was 10.0 (95% CI: 3.5–28.8; Table 1).

The prevalence of asymptomatic patent (microscopy-detectable) infections was 48.5% (16/33) in Cohort 1 compared with 12.0% (216/1807) in Cohort 2 (*P* < .0001); aOR = 5.7 (95% CI: 2.8–11.7; Table 1). The difference in subpatent (submicroscopic) infections between the two cohorts was not significant (*P* = .10; Table 1).

The prevalence of patent *P. falciparum* monoinfections was 30.3% (10/33) in Cohort 1, compared to 6.3% (114/1807) in Cohort 2 (*P* < .0001; Table 1). The corresponding prevalence of patent *P. vivax* was 15.2% (5/33) versus 4.8% (86/1807), respectively (*P* = .007; Table 1). The aORs for patent *P. falciparum* and patent *P. vivax* infections were 5.4 (95% CI: 2.5–11.9) and 3.2 (95% CI: 1.2–8.6), respectively (Table 1). The difference in prevalence of subpatent infections was not statistically significant for each species (*P* > .17; Table 1).

## DISCUSSION

We report population-based evidence that chronic infection with either *P. falciparum* or *P. vivax* is associated with splenic rupture and splenectomy after trauma. Our results highlight the very high prevalence (87.9%) of asymptomatic peripheral parasitemia among patients undergoing trauma-related splenectomy, which was more than twice the prevalence in the same general population. The relative difference in prevalence was the greatest in patent infections but was not significant in subpatent infections. The association was consistent for both *P. falciparum* and *P. vivax*, suggesting that chronic patent infection with either species may contribute to splenic vulnerability in endemic regions and may predispose the spleen to rupture.

Our findings point toward an additional important but under-recognized consequence of chronic asymptomatic malaria. In moderate to high transmission areas such as Timika where chronic infections are common, clinicians and public health practitioners should be aware that individuals with no overt malaria symptoms may still be harboring chronic infections that may place them at higher likelihood of splenic rupture, in addition to the broad range of other increasingly recognized long-term complications [4]. With patency being an associated factor, an adequate level of standard microscopy would identify and treat the majority of individuals that may be at risk.

Although splenic rupture has long been recognized as a complication of acute malaria [9, 10], our study suggests that chronic malaria may also present a significant risk of splenic complications. Notably, the absence of massive splenomegaly in most patients in our trauma-related splenectomy cohort (half <335 grams), only one of whom had hyperreactive malarial splenomegaly, suggests that even moderate degrees of splenomegaly in chronic malaria may portend clinically significant consequences. In chronic asymptomatic malaria, spontaneous or pathological rupture of the spleen is rarely described [12] and is thought to almost always be preceded by obvious trauma [10], consistent with our findings. In contrast to the rapid pathological changes seen in acute infections [9, 10], chronic exposure *to Plasmodium* is thought to result in more gradual splenic hyperplasia and red blood cell congestion and potentially a more gradual rise in intrasplenic tension [6]. This gradual pathogenesis may enable increased connective tissue formation and fibrosis [13], and thickening of the splenic capsule [14], ultimately resulting in a firm and often massively enlarged organ [10]. Our findings broadly align with this pathophysiological model highlighting that overt force is needed to cause initial capsular and subcapsular damage of the spleen in chronic malaria.

The likelihood that trauma-related splenic rupture may be greater in chronic patent infections may be explained by the relatively larger spleens in this group compared to subpatent infections, and thus, greater surface area exposed to trauma and a greater degree of cellular congestion and other structural changes described above. We speculate that the higher level of circulating parasitemia in patent infections may be exacerbating these processes, potentially through greater parasite-dependent and immune-related mechanisms reducing uninfected red cell deformability that underlies congestion [6, 15]. Fibrosis may also render the chronically infected spleen less amenable to undergo vasoconstriction in reaction to rupture, thereby increasing the risk of abundant bleeding requiring surgery.

Our study had several limitations. Firstly, given the absence of a trauma-exposed comparator group without splenic rupture, it was not possible to determine whether chronic malaria independently increases the risk of splenectomy following trauma. Our cross-sectional datasets also lacked longitudinal assessment of individuals with chronic malaria to quantitate the risk of splenic rupture over time. Secondly, the splenectomy cohort (Cohort 1) represents a small selected group of patients in whom spleen size and behavioral characteristics may not be representative of the broader chronically-infected population. We were unable to measure spleen size in the general population (Cohort 2), but the main determinant of spleen size in this setting is chronic malaria, which we were able to measure. In addition, our analyses show that differences between the two populations were not substantially affected when controlling for age and sex, which may serve as proxies for behavioral factors that may affect prevalence. Thirdly, the two cohorts were drawn from slightly different time periods (2013 vs 2015–2021), which could introduce temporal biases related to malaria transmission intensity. However, there was no increase in malaria incidence in 2015–21 relative to 2013 to account for this (Mimika District Health Authority, unpublished data). Mimika District Hospital is the sole public referral center for trauma, making it likely that our study captured the majority of cases of trauma-related splenectomy in this region. Lastly, causality cannot be definitively inferred from this observational design, though the consistency with the pathophysiological model may indicate a contributory role of chronic parasitemia in trauma-related splenic rupture requiring splenectomy.

In conclusion, our study provides comparative prevalence data indicating that patients undergoing trauma-related splenectomy are twice as likely to have chronic malaria than the general population and four times as likely to have patent infections, suggesting that chronic *P. falciparum* or *P. vivax* infection may predispose individuals to splenic rupture following trauma. Whether chronic malaria is an independent risk factor for trauma-related splenic rupture deserves further study. Our findings underscore the importance of recognizing chronic malaria as not benign, and a condition carrying multiple under-recognized risks for sequelae beyond anemia and febrile illness. In endemic settings, malaria control and elimination efforts should be prioritized not only to prevent acute disease but also to mitigate the full range of long-term complications affecting individuals with chronic subclinical infections, that now may include splenic rupture.

## References

[1] Poespoprodjo JR, Douglas NM, Ansong D, Kho S, Anstey NM. Malaria. Lancet 2023; 402:2328–45. 37924827 10.1016/S0140-6736(23)01249-7

[2] Tadesse FG, Slater HC, Chali W, et al The relative contribution of symptomatic and asymptomatic plasmodium vivax and plasmodium falciparum infections to the infectious reservoir in a low-endemic setting in Ethiopia. Clin Infect Dis 2018; 66:1883–91. 29304258 10.1093/cid/cix1123

[3] White NJ, Pukrittayakamee S, Hien TT, Faiz MA, Mokuolu OA, Dondorp AM. Malaria. Lancet 2014; 383:723–35. 23953767 10.1016/S0140-6736(13)60024-0

[4] Chen I, Clarke SE, Gosling R, et al “Asymptomatic” malaria: a chronic and debilitating infection that should be treated. PLoS Med 2016; 13:e1001942. 26783752 10.1371/journal.pmed.1001942 PMC4718522

[5] Douglas NM, Lampah DA, Kenangalem E, et al Major burden of severe anemia from non-falciparum malaria species in Southern Papua: a hospital-based surveillance study. PLoS Med 2013; 10:e1001575. 24358031 10.1371/journal.pmed.1001575 PMC3866090

[6] Kho S, Siregar NC, Qotrunnada L, et al Retention of uninfected red blood cells causing congestive splenomegaly is the major mechanism of anemia in malaria. Am J Hematol 2024; 99:223–35. 38009287 10.1002/ajh.27152 PMC10952982

[7] Kho S, Qotrunnada L, Leonardo L, et al Hidden biomass of intact malaria parasites in the human spleen. N Engl J Med 2021; 384:2067–9. 34042394 10.1056/NEJMc2023884

[8] Kho S, Qotrunnada L, Leonardo L, et al Evaluation of splenic accumulation and colocalization of immature reticulocytes and *Plasmodium vivax* in asymptomatic malaria: a prospective human splenectomy study. PLoS Med 2021; 18:e1003632. 34038413 10.1371/journal.pmed.1003632 PMC8154101

[9] Imbert P, Rapp C, Buffet PA. Pathological rupture of the spleen in malaria: analysis of 55 cases (1958–2008). Travel Med Infect Dis 2009; 7:147–59. 19411041 10.1016/j.tmaid.2009.01.002

[10] Zingman BS, Viner BL. Splenic complications in malaria: case report and review. Clin Infect Dis 1993; 16:223–32. 8443301 10.1093/clind/16.2.223

[11] Pava Z, Burdam FH, Handayuni I, et al Submicroscopic and asymptomatic *Plasmodium* parasitaemia associated with significant risk of Anaemia in Papua, Indonesia. PLoS One 2016; 11:e0165340. 27788243 10.1371/journal.pone.0165340 PMC5082812

[12] Tola GG, Hemba YG, Hussen SK, Matiop MP. Case series of spontaneous splenic rupture in patients with malaria: a rare surgical emergency in non-traumatic cases. Int J Surg Case Rep 2025; 134:111712. 40714671 10.1016/j.ijscr.2025.111712 PMC12318301

[13] Wanless IR, Bernier V. Fibrous thickening of the splenic capsule. A response to chronic splenic congestion. Arch Pathol Lab Med 1983; 107:595–9. 6688717

[14] Mabogunje OA . Conservation of the ruptured spleen in children: an African series. Ann Trop Paediatr 1990; 10:387–93. 1708968 10.1080/02724936.1990.11747463

[15] Oyong DA, Kenangalem E, Poespoprodjo JR, et al Loss of complement regulatory proteins on uninfected erythrocytes in vivax and falciparum malaria anemia. JCI Insight 2018; 3:e124854. 30429373 10.1172/jci.insight.124854 PMC6303009

